# Complete Genome Sequence of the Plasmid-Bearing *Lactobacillus fermentum* Strain SNUV175, a Probiotic for Women’s Health Isolated from the Vagina of a Healthy South Korean Woman

**DOI:** 10.1128/genomeA.00045-17

**Published:** 2017-03-23

**Authors:** Sunghee Lee, Hyun Ju You, Bomi Kwon, GwangPyo Ko

**Affiliations:** a Center for Microbiome Therapeutics, KoBioLabs, Inc., Seoul, Republic of Korea; b N-Bio, Seoul National University, Seoul, Republic of Korea; c Department of Environmental Health Sciences, Graduate School of Public Health, Seoul National University, Seoul, Republic of Korea; d Institute of Health and Environment, Seoul National University, Seoul, Republic of Korea; e Center for Human and Environmental Microbiome, Seoul National University, Seoul, Republic of Korea

## Abstract

*Lactobacillus fermentum* SNUV175 has been identified as a probiotic strain that inhibits pathogenic microorganisms related to women’s health. We present the complete genomic sequence of the strain *L. fermentum* SNUV175 isolated from the vagina of a South Korean woman. This genomic information may provide insight into the functional activity of this strain.

## GENOME ANNOUNCEMENT

The composition of the vaginal microbiota is influenced by host genetics and plays a key role in host defense against bacterial vaginosis and pathogenic viral infections, such as human papillomavirus (HPV) and human immunodeficiency virus (HIV) (1–3). Among the microorganisms present in the vagina, *Lactobacillus* spp. help maintain an acidic environment and produce bacteriocins that inhibit potentially pathogenic microorganisms (4).

In this study, the strain *Lactobacillus fermentum* SNUV175, isolated from the vagina of a healthy South Korean woman, showed functional properties in the treatment of vaginal infection caused by lactobacilli deficiency. Here, we present the complete genome sequence of this functional probiotic strain.

In order to perform the complete genome sequencing of the strain *L. fermentum* SNUV175, massive sequencing technology was implemented using the PacBio platform (Pacific Biosciences, Menlo Park, CA, USA). A 20-kb library was constructed with purified DNA on a single-molecule real-time (SMRT) cell and was sequenced using P6-C4 chemistry with a data collection time of 4 h. The sequencing run provided a total of 125,532 reads with quality scores ≥Q20. The number of bases was 805,055,752 bp. *De novo* assembly employed the default parameters of the Hierarchical Genome Assembly Process approach version 3 (HGAP3) (5). The single circular chromosome was 2,176,678 bp in size, with an estimated G+C content of 51.5% and around 276× coverage. The three plasmids ranged between 29 and 33 kb in size: pSNU175-2 (33,413 bp; G+C content, 40.0%), pSNU175-3 (33,071 bp; G+C content, 39.6%), and pSNU175-3 (29,166 bp; G+C content, 43.8%).

The assembled genome sequences were annotated using the Prokka annotation pipeline, version 1.11 (6), which predicted tRNA, rRNA, and mRNA genes. Putative gene products were then assigned to protein-coding genes (CDSs) based on their similarity to sequences in the respective database. Curated virulence factors and antibiotic resistance genes were estimated using IslandViewer3 (7) against the Virulence Factor Database (VFDB) (8) and the Comprehensive Antibiotic Resistance Database (CARD) (9).

The genome contains 2,272 CDSs, 59 tRNAs, and 15 rRNAs. The *L. fermentum* SNUV175 genome was compared with reference strain *L. fermentum* IFO3956 (GenBank accession no. AP008937.1) using the Rapid Annotations using Subsystems Technology (RAST) server (10). Through this comparison we detected 116 elements not present in the published strain *L. fermentum* IFO3956. No remarkable antibiotic resistance or virulence-associated genes were found. The analysis of the complete genome of *L. fermentum* SNUV175 may lead to deeper understanding of the mechanisms involved in its effect against bacterial vaginosis.

### Accession number(s).

The results of this whole-genome project have been deposited at GenBank under the accession numbers CP019030 to CP019033.
